# On-chip passive three-port circuit of all-optical ordered-route transmission

**DOI:** 10.1038/srep10190

**Published:** 2015-05-13

**Authors:** Li Liu, Jianji Dong, Dingshan Gao, Aoling Zheng, Xinliang Zhang

**Affiliations:** 1 Wuhan National Laboratory for Optoelectronics, Huazhong University of Science and Technology, Wuhan, 430074 China; 2 State Key Laboratory on Integrated Optoelectronics, College of Electronic Science and Engineering, Jilin University, Changchun, 130012 China

## Abstract

On-chip photonic circuits of different specific functions are highly desirable and becoming significant demands in all-optical communication network. Especially, the function to control the transmission directions of the optical signals in integrated circuits is a fundamental research. Previous schemes, such as on-chip optical circulators, are mostly realized by Faraday effect which suffers from material incompatibilities between semiconductors and magneto-optical materials. Achieving highly functional circuits in which light circulates in a particular direction with satisfied performances are still difficult in pure silicon photonics platform. Here, we propose and experimentally demonstrate a three-port passive device supporting optical ordered-route transmission based on silicon thermo-optic effect for the first time. By injecting strong power from only one port, the light could transmit through the three ports in a strict order (1→2, 2→3, 3→1) while be blocked in the opposite order (1→3, 3→2, 2→1). The blocking extinction ratios and operation bandwidths have been investigated in this paper. Moreover, with compact size, economic fabrication process and great extensibility, this proposed photonic integrated circuit is competitive to be applied in on-chip all-optical information processing systems, such as path priority selector.

Nonreciprocal optical devices, which could control the transmission directions of the optical signals are fundamental researches in optical communication systems^1^,^2^,^3^,^4^,^5^,^6^,^7^. Especially, highly functional circuits, such as optical circulators^8^,^9^, in which light circulates in a particular direction are very important for practical applications^10^,^11^,^12^. Currently, commercially available such optical circuits consist of several bulk components^13^,^14^, such as Faraday rotators, polarization beam splitters and half-wave plates. To miniaturize the device size, efforts have focused on ultra-compact on-chip counterparts^15^,^16^,^17^. To date, only several works have experimentally demonstrated these functional circuits^18^,^19^ while most schemes still step at the states of theoretical simulations^20^,^21^,^22^. Besides, the fabricated devices in refs. 18 and 19 are based on the magneto-optical materials which are complex to operate and incompatibility with complementary metal-oxide semiconductor (CMOS).

Due to the dominant advantages of silicon-on-insulator (SOI) technology, such as CMOS-compatible and high refractive index difference, integrated circuits based on silicon nano-photonic technology are promising to become the mainstay of the photonics industry^23^,^24^,^25^. All-optical ordered-route transmission means that light circulates in a particular ordered direction which is significant in practical applications. However, it is still challenging to realize this function on pure silicon platform. Toward the large-scale integration and low power consumption, the requirement to overcome this difficulty becomes urgent.

In this letter, we for the first time experimentally demonstrate a three-port passive device of all-optical ordered-route transmission on pure silicon platform. It means that the light can transmit from one port to another with a strict order (such as Port 1→ Port 2, Port 2→ Port 3, Port 3→ Port 1), but will be blocked in the opposite order. The operation principle for this device is the thermo-optic effect in silicon microring resonators (MRRs) whose transmission spectra could be shifted by injecting strong light power^26^.

## Results

### Theoretical analysis

The physical mechanism of our scheme is the thermo-optic effect of silicon. With an asymmetrically coupled MRR, Fan *et al.* has demonstrated that for identical optical power injection, the power inside the MRR is higher when light is injected from a bus waveguide with a strong coupling than the other side with a weak coupling^26^,^27^. When the power inside the MRR accumulates high enough, the thermo-optic effect dominates the free-carrier nonlinear effect thus leading to a red-shift of the resonance wavelength^28^.

**Figure 1:**
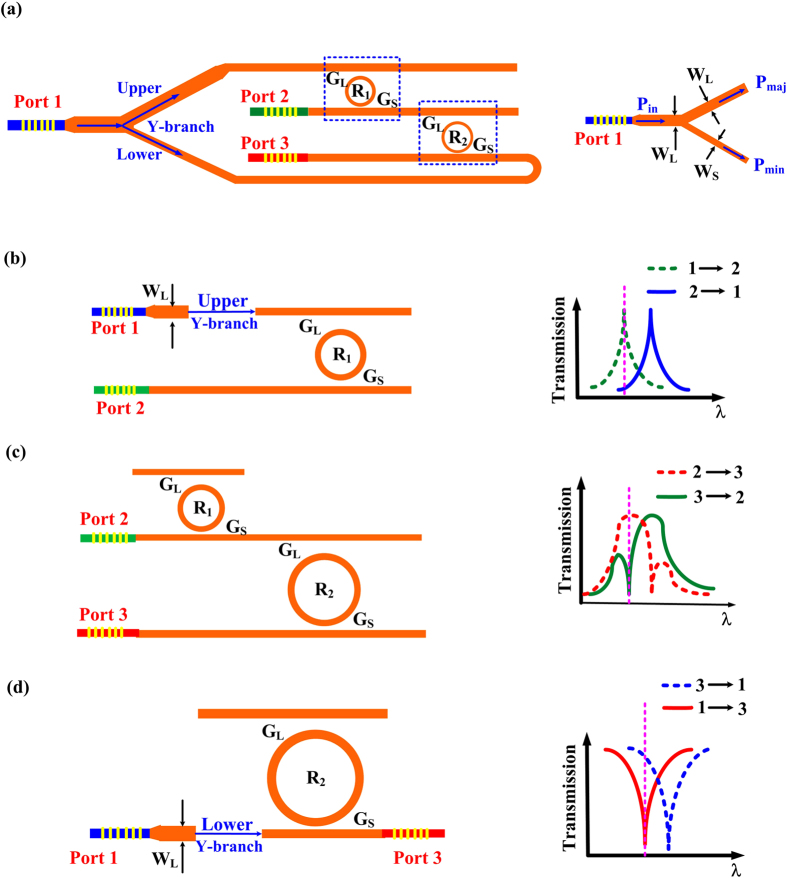
Theoretical analysis of the three-port device. **(a)** Layout of the proposed three-port device of optical ordered-route transmission. **(b)**-**(d)** Principle diagrams of route-asymmetric optical transmission between **(b)** Port 1 and Port 2, **(c)** Port 2 and Port 3, **(d)** Port 3 and Port 1, respectively. All input light own the same power at an aligned resonant wavelength of the two MRRs. Abbreviations are labelled as follows: P_in_, input power; P_maj_, major power; P_min_, minor power; W_L_, larger width; W_S_, smaller width; G_L_, larger gap; G_S_, smaller gap.

The layout of the proposed device is shown in Fig. 1(a). It mainly consists of a Y-branch and two asymmetric add-drop MRRs (named as R_1_ and R_2_), with each having different gaps (named as G_L_ and G_S_) between the straight waveguides and resonators, respectively. G_L_ is much larger than G_S_ in order to realize a weaker coupling and the resonant wavelengths of the two MRRs should be designed identically. In addition, the waveguide widths (W_L_ and W_S_) of the Y-branch are different to split the input power unequally. The major power (P_maj_) of the light injected from Port 1 (P_in_) propagates in the upper arm of the Y-branch because of a larger waveguide width (W_L_). Meanwhile, the minor power (P_min_) transmits through the lower arm due to a smaller waveguide width (W_S_). Figs. 1(b)–(d) show the detailed descriptions of unidirectional transmission for each pair of ports.

As shown in Fig. 1(b), assume that a strong light is injected into Port 1, whose wavelength is an aligned resonance of the two MRRs. Although the major light (P_maj_) passes through the upper arm of the Y-branch and arrives at R_1_, only a weak energy couples into the microring due to the larger gap of G_L_, which is not high enough to cause an effective red-shift of the transmission spectrum, thereby keeping R_1_ at cold resonance. Thus there is a maximum transmission at the input wavelength from Port 1 to Port 2 (green dashed line). In contrast, when the input light with the same optical power and wavelength is loaded at Port 2, due to the small gap of G_S_ there is a strong coupling energy stored in R_1_ with a hot resonance leading to an appreciable red-shift. Thus there is a minimum transmission from Port 2 to Port 1 (blue solid line). Therefore, by launching a high-power light with the initial resonant wavelength, we can realize a unidirectional transmission between Port 1 and Port 2. Namely, the light can transmit from port1 to port 2 with a certain waveguide loss but the opposite direction is blocked.

Figure 1(c) presents the unidirectional transmission between Port 2 and Port 3. Obviously, since the two MRRs of R_1_ and R_2_ are cascaded, the total transmission spectrum is the multiplication of the two MRRs with one at the through-port of R_1_ and the other at the drop-port of R_2_. When the strong power is launched into Port 2, the spectral notch of R_1_ will be red shifted due to hot resonance (smaller gap of G_S_), while the spectral peak of R_2_ keeps relatively stable due to cold resonance (larger gap of G_L_). Thus the total transmission spectrum at Port 3 is shown as the red dashed line. However, when the strong power is launched into Port 3, the spectral peak of R_2_ will be obviously red shifted due to the smaller gap of G_S_, but the spectral notch of R_1_ will keep almost stable because very few light power could drop from R_2_ and arrive at R_1_. Thus the total output spectrum at Port 2 is shown as the green solid line. Therefore, due to the asymmetric red-shift of R_1_ and R_2_ respectively, the light can transmit from Port 2 to Port 3 but the opposite direction is cut-off.

Figure 1(d) shows the unidirectional transmission between Port 3 and Port 1 whose principle is similar to Fig. 1(b). As we launch the light into Port 3 with the same input condition, the transmission spectrum of R_2_ experiences a significant red shift, leading to a large transmission from Port 3 to Port 1. While for the opposite transmission, only a minor power (P_min_) of the input power (P_in_) propagates in the lower arm of the Y-branch because of a smaller waveguide width. Hence, the power is not strong enough to cause an effective red-shift of R_2_, leading to a light block from Port 1 to Port 3. Hence, the light can transmit from Port 3 to Port 1 (blue dashed line) while the opposite direction is turned off (red solid line).

Therefore, the light could transmit in a specific ordered route when only one port is injected of a high-power light, namely from Port 1 to Port 2, Port 2 to Port 3, Port 3 to Port 1 (1→2, 2→3, 3→1) while the opposite transmissions (1→3, 3→2, 2→1) are blocked. It should be noted that a significant feature of the proposed device is the extensible topological structure with the basic unit of a MRR with different gaps at the through and drop ports respectively, as shown in the blue boxes in Fig. 1(a). Hence, to meet the practical communication requirements for large-scale integrated circuit, more multi-port devices of all-optical ordered-route transmission could be simply achieved by combining different number of the basic units.

### Device structure

**Figure 2:**
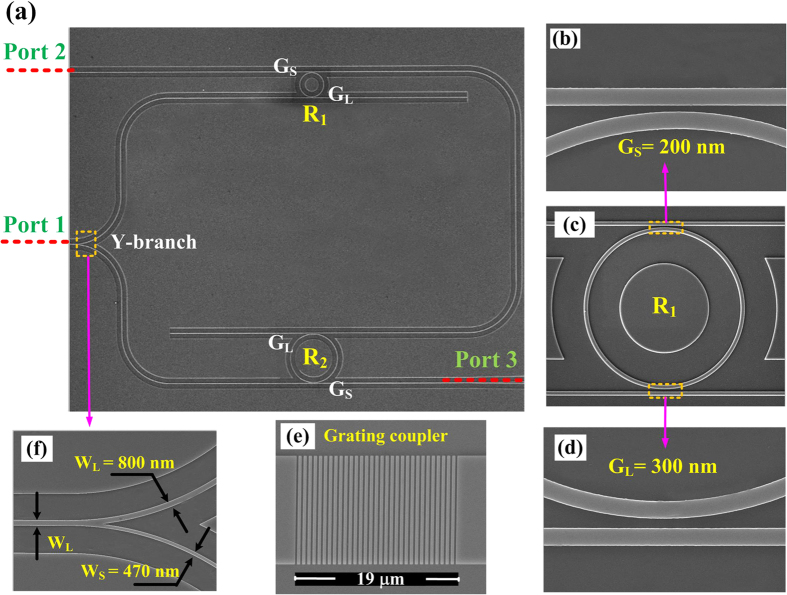
SEM images. **(a)** SEM image of the three-port device. The radii of the two MRRs (R_1_, R_2_) are 10 μm and 20 μm. The waveguide widths of both bus waveguide and bending waveguide are about 500 nm. The zoom in figures of **(b)** and **(d)** are the coupling regions of drop port and through port of R_1_ **(c)** respectively. The coupling gaps of G_L_ and G_S_ are 300 nm and 200 nm respectively. **(e)** and **(f)** are the zoom in images of the grating coupler and Y-branch, respectively.

Theoretically, the two MRRs with the same radius can also be feasible to meet our requirements. However, without a tuning technique, it is difficult to precisely match their spectra due to fabrication imperfection. Therefore, two MRRs with different radii are designed in our experiment. Due to the Vernier effect, there are always several peaks of the two cascaded MRRs that perfectly match with a certain free spectral range (FSR). We first design and fabricate the device on a commercial SOI wafer. Fig. 2(a) shows the scanning electron microscope (SEM) image of the device consisting of a Y-branch and two MRRs with different radii (R_1 _= 10 μm, R_2 _= 20 μm). The coupling gaps of G_L_ and G_S_ for R_1_ in Fig. 2(c) are 300 nm and 200 nm whose zoom in images are shown in Figs. 2(b) respectively. The coupling gaps of G_L_ and G_S_ for R_2_ are set the same with those of R_1_. We employ the vertical grating coupler to couple the optical signal from fiber to silicon waveguide, and the zoom in grating coupler is shown in Fig. 2(e). The period, duty cycle, total length, 3-dB coupling bandwidth and coupling loss for a single side of the grating coupler are 630 nm, 56%, 19 μm, 30 nm and 6 dB, respectively. The waveguide widths of W_L_ and W_S_ of the Y-branch are 800 nm and 470 nm respectively, as shown in Fig. 2(f).

**Figure 3:**
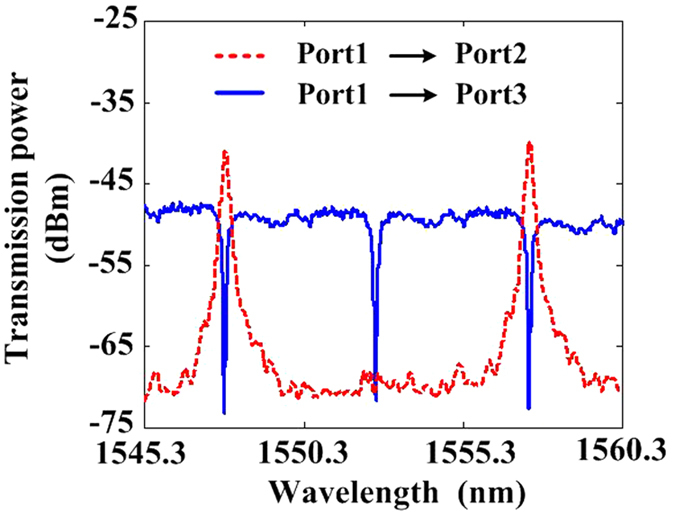
Measured transmission spectra. Measured transmission spectra from Port 1 to Port 2 (red dashed line) and Port 1 to Port 3 (blue solid line). In the two lines, there are two aligned resonant wavelengths of 1547.72 nm and 1557.35 nm.

Figure 3 shows the measured transmission spectra from Port 1 to Port 2 (red dashed line) and Port 1 to Port 3 (blue solid line) which correspond to the drop-port spectrum of R_1_ and through-port spectrum of R_2_, respectively. The FSRs of R_1_ and R_2_ are about 9.63 nm and 4.81 nm. Obviously, there are some aligned resonant wavelengths with a FSR of 9.63 nm, such as 1547.72 nm and 1557.35 nm in the two lines. The insertion losses are around 6 dB (between Port 1 and Port 2), 5 dB (between Port 2 and Port 3) and 11 dB (between Port 1 and Port 3) after subtracting the 12 dB fibre-chip-fibre coupling loss, respectively.

### Experiment overview

**Figure 4:**
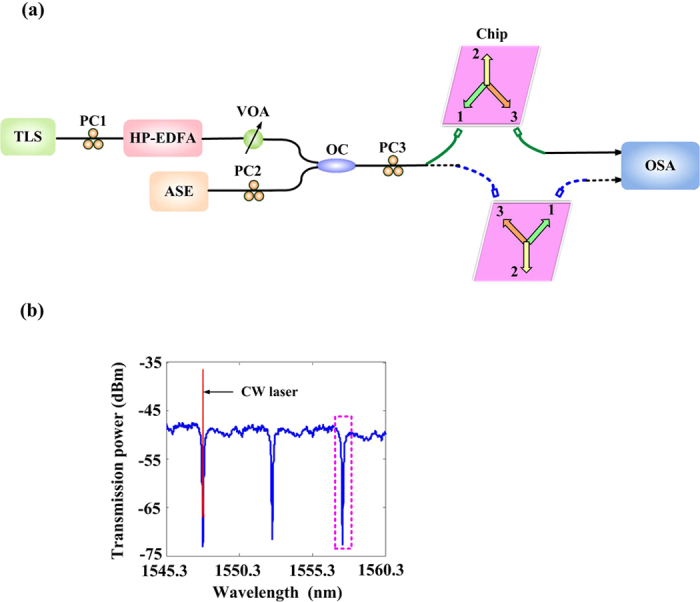
Experimental setup and measured regions. **(a)** Schematic diagram of the experimental setup. The components of the apparatus are labelled as follows: TLS, tunable laser source; PC, polarization controller; HP-EDFA, high-power erbium-doped fiber amplifier; VOA, variable optical attenuator; ASE, amplified Spontaneous Emission; OC, optical coupler; OSA, optical spectrum analyzer. **(b)** Measured regions of the laser powers and ASE red-shifts. The laser powers and the red-shift ASE spectra are measured around 1547.72 nm (the red line) and around 1557.35 nm (the pink box), respectively.

In order to verify the above theoretical analysis, we carry out an experiment as shown in Fig. 4(a). First, we tune the output power and wavelength of a continuous wave (CW) light emitted from a tunable laser source (TLS) at 10 dBm. Then we adjust a high-power erbium-doped fiber amplifier (HP-EDFA) to amplify the output power to fix at 21 dBm. In fact, an optical power of ~15 dBm is injected into each port of the device in consideration of 6-dB fiber-to-chip loss. A low-power amplified spontaneous emission (ASE) source is coupled with the input CW light by an optical coupler (OC) and injected to the silicon device by the vertical coupling system to characterize the transmission spectrum.

The CW wavelength is aligned to the MRR resonant wavelength, i.e., 1547.72 nm which is shown as the red line in Fig. 4(b). Due to the existence of ASE source, red-shifts can be measured conveniently by analyzing the spectrum around another resonant wavelength, i.e., 1557.35 nm which is shown as the pink box. In this way, we can accurately measure both the laser powers (around 1547.72 nm) and red-shifts of the MRR spectrum (around 1557.35 nm).

### Experimental results

**Figure 5:**
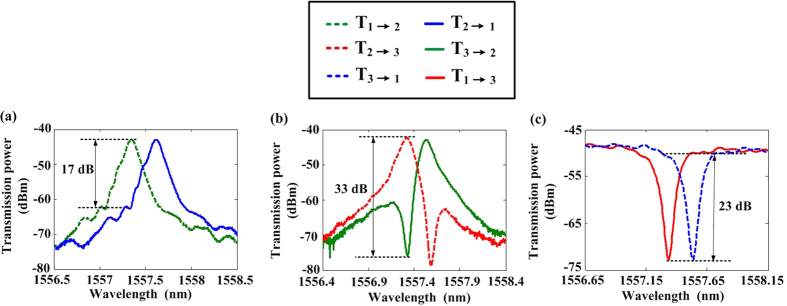
Measured red-shift spectra. Measured ASE spectra of route-asymmetric transmission between **(a)** Port 1 and Port 2, **(b)** Port 2 and Port 3, **(c)** Port 3 and Port 1, respectively. For all images, input laser power into each port is 15 dBm at the wavelength of 1547.72 nm.

Figure 5(a)–(c) show the measured transmission spectra corresponding to the schematic transmission spectra in Figs. 1(b)–(d), respectively. The transmittance spectra of 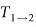 (from Port 1 to Port 2, green dashed line) and 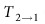 (from Port 2 to Port 1, blue solid line) are measured asynchronously as shown in Fig. 5(a). The blocking extinction ratio (BER) is defined as the difference (in dB unit) of the two transmittance spectra (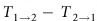) at the input wavelength. There is a distinct red-shift of 0.23 nm between the two transmittance spectra which results in a BER of 17.3 dB at 1557.35 nm. Then, we have also measured the transmittance spectra of 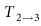 (from Port 2 to Port 3, red dashed line) and 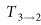 (from Port 3 to Port 2, green solid line) as shown in Fig. 5(b). The BER of 33 dB (
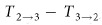
) indicates that the path from Port 3 to Port 2 is almost blocked. Fig. 5(c) presents the unidirectional transmission results between Port 3 and port 1. The measured transmittance spectra of 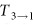 (from Port 3 to Port 1) and 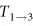 (from Port 1 to Port 3) are shown as the red solid line and blue dashed line, respectively. Obviously, the red-shift and BER are 0.2 nm and 23 dB (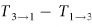). By injecting light from only one port of the device, we have demonstrated the desired three-port device of optical ordered-route transmission (1→2, 2→3, 3→1), but the opposite transmissions (1→3, 3→2, 2→1) are blocked.

**Figure 6:**
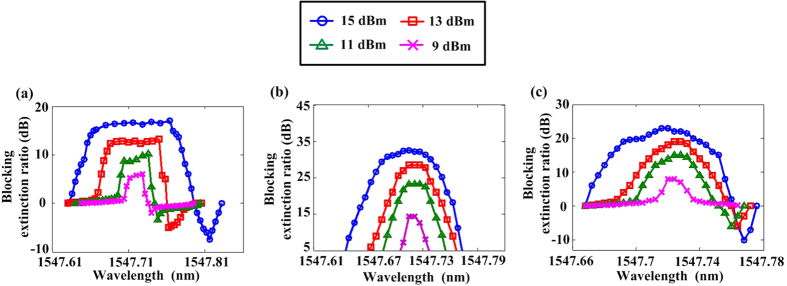
Experimental results under different power inputs. Route-asymmetric performances of **(a)** Port 1 and Port 2, **(b)** Port 2 and Port 3, **(c)** Port 3 and Port 1, respectively.

To further characterize the unidirectional transmission performance, we have measured the operation bandwidth (BW) of the proposed device. The input powers are set at 15 dBm, 13 dBm, 11 dBm and 9 dBm respectively and we repeat the measurements. Figs. 6(a)–(c) describe the variation of the BERs between each pair of ports respectively (corresponding to Port 1 and Port 2, Port 2 and Port 3, Port 3 and Port 1), as the wavelength is tuned from 1547.62 nm to 1547.82 nm. Table 1 summarizes the measured unidirectional transmission results between every two ports of the device under different input powers. The 15-dB BW represents the operation bandwidth with BER larger than 15 dB. When the input power is set at 15 dBm for each port, the BER and 15-dB BW of the whole device are larger than 17 dB and 0.08 nm, respectively. Obviously, both the BER and BW can be improved as the input power increases, which indicate that the performance of the three-port device is controllable by the injected power.

### Practical application for path priority selector

**Figure 7:**
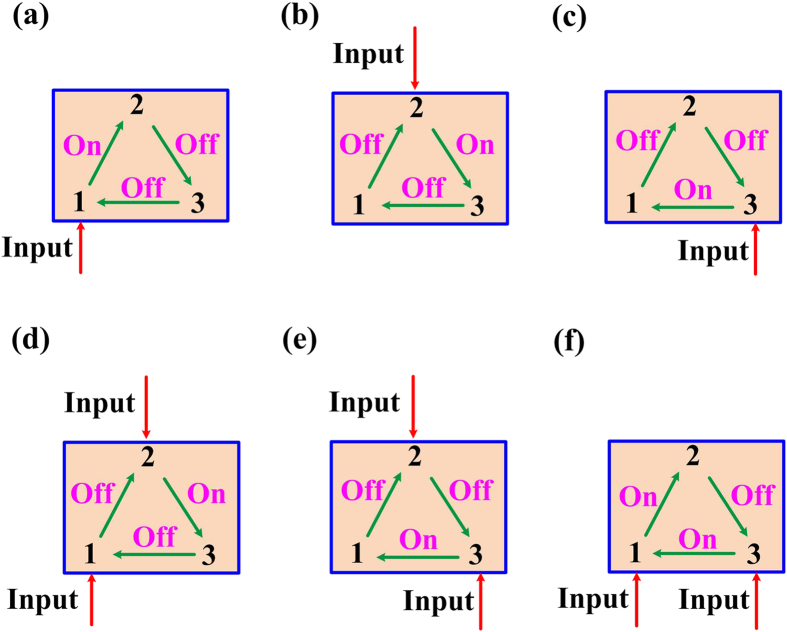
Path priority selector. The green and red arrows indicate the transmission routes and input ports of strong power, respectively. Marks of on and off stand for the working states of the three routes (1→2, 2→3, 3→1) with input at (**a**) only Port 1, (**b**) only Port 2, (**c**) only Port 3, (**d**) Port 1 and Port 2, (**e**) Port 2 and Port 3, (**f**) Port 1 and Port 3, respectively.

In this paper, our main target is to design a three-port passive device of all-optical ordered-route transmission, which is similar to the optical circulators. The experiment demonstrated that this device could achieve unidirectional propagation depending on the input power delivered at each circuit port as shown in Figs. 7(a)–(c). Besides the function of ordered-route transmission, our device has some unique applications that optical circulators could not do in all-optical network, such as path priority selector as shown in Figs. 7(d)–(f). Without loss of generality, we consider three signal paths (1→2, 2→3, 3→1). As shown in Fig. 7(a), when a signal with a strong power is injected from only Port 1, the signal could route to Port 2, thus Path I (from Port1 to Port 2) is activated. Fig. 7(d) shows that as long as Port 2 accepts a high power injection, Path II (from Port 2 to Port 3) is activated, while Path I is immediately quenched. In this case, due to the high power injection, the spectrum of R_1_ is red shifted to block the light in path I while activate path II. We know that when there is a competition of signal transmission between Port 1 and 2, Port 2 is prior. Meanwhile, Fig. 7(e) shows that when Port 2 and 3 are simultaneously injected by a strong power, Path II is quenched while Path III (from Port 3 to Port 1) is activated. In this case, because of the high power injection, the spectrum of R_2_ is red shifted to block the light in path II while activate path III. Hence, when there is a competition between Port 2 and 3, Port 3 is prior which can be concluded from Figs. 7(b). Note that Port 1 and Port 3 act as the beginning port and ending port of the circuit respectively, thus they have the same priority if there is a comptition, as shown in Fig. 7(f). Therefore, the proposed structure appoints that when adjacent ports have input signals simultaneously, the port with higher priority is prior to transmit signals. This function can alleviate the problem of data competition in time sequence.

## Discussion

All-optical ordered-route transmission is important in optical communication network. However, until now, it is still difficult to realize this function in pure silicon platform. In this study, we have proposed a compact optical circuit using two silicon MRRs, as shown in Fig. 2. This three-port passive device based on thermo-optic effect could support all-optical ordered-route transmission with satisfied performances. By injecting from only one port, the light of strong power could transmit through the three ports in a specific order (1→2, 2→3, 3→1) while the opposite order is blocked. As shown in Table 1, The BER and 15-dB BW of the whole device are larger than 17 dB and 0.08 nm, respectively. Among the performances of every two ports, a maximum BER up to 33 dB has been obtained, as well as a relatively broad 15-dB BW of 0.1 nm.

It should be noted that the performance of this device has lots of potential improvements in the future. First, by designing the MRRs at the critical coupling and accurately aligning the mismatch resonant wavelengths of the two MRRs by the micro-heater, the maximum BER can be improved. In ref. 27, a maximum BER of 40 dB was achieved. Second, the injection power and insertion loss can be reduced greatly by utilizing a better grating coupler design^29^ and ultralow-loss waveguide fabrication^30^,^31^ to make this device suitable for large-scale integrated circuits. If we use the grating coupler of −0.58 dB coupling efficiency^29^, the microring resonator of 2-3 dB/cm transmission loss^30^ and the optical waveguides of 0.026 dB/cm loss^31^, the input power can be significantly reduced. Fan *et al.* achieved the maximum BER of 40 dB at ~2.3 mW^27^. Furthermore, because of the small mode volumes of photonic crystal nano-cavities, the operation powers to activate these nano-cavities are much lower than microring-based systems^32^. Thus we could instead the microring resonators of photonic crystal nano-cavities to further decrease the input power.

The thermo-optic effect to realize optical isolation has an limited bandwidth, which is the intrinsic characteristic of high-Q resonant devices^26^,^27^,^28^,^32^. However, we could improve this shortcoming by using the micro-heaters to shift the resonant wavelengths of the resonators according to the operation wavelength. Thus the locations of the operation bandwidth could be tunable to meet the practical demands in optical communication systems.

Although we only demonstrate a three-port circuit, our designed structure is extensible. As shown in Fig. 1(a), the basic unit is an asymmetrically coupled MRR, so multi-port optical circuits could be realized through composing different number of the basic units in the future. Moreover, besides the function of ordered-route transmission, our device has many other significant applications in all-optical network, such as path priority selector as shown in Fig. 7. This function can solve the problem of data competition in time sequence.

In summary, we have for the first time experimentally demonstrated a three-port passive device supporting all-optical ordered-route transmission structure based on thermo-optic effect in silicon MRRs. The experimental performances show that it can works well to control the optical transmission directions. This integrated and extensible photonic circuit which doesn’t require extra assistance such as magnetic fields, time-space modulation, or external optical pumping, is promising to find significant applications in all-optical communication network.

## Methods

### Devices fabrication

The top silicon thickness of the SOI wafer is 340 nm, and the buried oxide layer thickness is 2 μm. The device layout was transferred to ZEP520A photoresist by E-beam lithography (Vistec EBPG5000+ES). Then, the upper silicon layer was etched downward for 240 nm to form a ridge waveguide through inductively coupled plasma (ICP) etching (Oxford Instruments Plasmalab System100).

## Author Contributions

J.J.D. and L.L. conceived the study. L.L. and X.L.Z. fabricated the device. L.L., D.S.G and A.L.Z. carried out the experiment. L.L. analyzed the data and wrote the manuscript. J.J.D. supervised the project and edited the manuscript. All authors discussed the results and commented on the manuscript.

## Additional Information

**How to cite this article**: Liu, L. *et al*. On-chip passive three-port circuit of all-optical ordered-route transmission. *Sci. Rep.* **5**, 10190; doi: 10.1038/srep10190 (2015).

## Figures and Tables

**Table 1:** Experimental results of the device.

**P**_**in**_ **(dBm)**	**Ports**	**BER (dB)**	**15-dB BW (nm)**
15	1 , 2	17.3	0.09
	2 , 3	33	0.1
	3 , 1	23	0.084
13	1 , 2	12.5	—
	2 , 3	27	0.06
	3 , 1	19	0.04
11	1 , 2	10.5	—
	2 , 3	23	0.04
	3 , 1	15	0.02
